# A review of the cognitive impact of neurodevelopmental and neuropsychiatric associated copy number variants

**DOI:** 10.1038/s41398-023-02421-6

**Published:** 2023-04-08

**Authors:** Ciara J. Molloy, Ciara Quigley, Áine McNicholas, Linda Lisanti, Louise Gallagher

**Affiliations:** 1 Department of Psychiatry, School of Medicine, Trinity College Dublin, Dublin, Ireland; 2 Trinity Centre for Health Sciences, St. James’s Hospital, Dublin, Ireland; 3 The Hospital for SickKids, Toronto, ON Canada; 4 The Peter Gilgan Centre for Research and Learning, SickKids Research Institute, SickKids Research Institute, Toronto, ON Canada; 5 The Centre for Addiction and Mental Health, Toronto, ON Canada; 6 Department of Psychiatry, Temerty Faculty of Medicine, University of Toronto, Toronto, ON Canada

**Keywords:** Clinical genetics, Human behaviour

## Abstract

The heritability of intelligence or general cognitive ability is estimated at 41% and 66% in children and adults respectively. Many rare copy number variants are associated with neurodevelopmental and neuropsychiatric conditions (ND-CNV), including schizophrenia and autism spectrum disorders, and may contribute to the observed variability in cognitive ability. Here, we reviewed studies of intelligence quotient or cognitive function in ND-CNV carriers, from both general population and clinical cohorts, to understand the cognitive impact of ND-CNV in both contexts and identify potential genotype-specific cognitive phenotypes. We reviewed aggregate studies of sets ND-CNV broadly linked to neurodevelopmental and neuropsychiatric conditions, and genotype-first studies of a subset of 12 ND-CNV robustly associated with schizophrenia and autism. Cognitive impacts were observed across ND-CNV in both general population and clinical cohorts, with reports of phenotypic heterogeneity. Evidence for ND-CNV-specific impacts were limited by a small number of studies and samples sizes. A comprehensive understanding of the cognitive impact of ND-CNVs would be clinically informative and could identify potential educational needs for ND-CNV carriers. This could improve genetic counselling for families impacted by ND-CNV, and clinical outcomes for those with complex needs.

## Introduction

Heritability estimates for general cognitive ability (or intelligence) range from 41% in childhood to 66% in adults [1]. Different forms of genetic variation, common and rare, may contribute to cognition, such as loss of function or missense single nucleotide variants (SNV), single nucleotide polymorphisms (SNP), and copy number variants (CNV). To date 11,600 SNPs in 148 loci have been linked to cognitive ability, however each have very small effects [2]. Polygenic scores, which are the weighted sum of trait associated variants, account for only 4.3% of variance in cognition [2]. Cognitive ability may be further accounted for by rare structural variants of larger effect, such as CNV [3]. CNV refer to variation in chromosomal copy typically leading to a loss (deletion) or gain (duplication) of sections of DNA that may include one or many genes. They can be common or rare, defined based on their frequency in the population and may be inherited or arise de novo in the germline. While CNV are commonly found in the general population and often benign, they can also be pathogenic. There has been particular interest in investigating rare CNV in human health and disease since these are likely more deleterious due to increased selection pressure [4]. Recurrent CNV are deletions or duplications that occur at specific genomic regions frequently associated with incomplete penetrance and variable expressivity. Although their phenotypic impact is not fully understood, many rare recurrent CNV have been linked to neurodevelopmental and neuropsychiatric conditions (ND-CNV) and contribute to associated traits such as variability in cognitive ability.

## CNV burden and effect sizes of deletions and duplications on cognition

Many CNV studies of cognition have focused on total CNV burden, defined as total length or number of base pairs impacted by a CNV [5, 6], and also numbers of affected genes in some studies [5, 7]. These studies examine unfiltered CNVs (i.e., they do not filter for neurodevelopmental-related CNV). Deletions and duplications are assessed either together or separately. CNV burden studies show mixed findings. Multiple general population studies suggest no effect of rare CNV burden, total number of CNV, or number of affected genes on general cognitive ability [3, 8, 9]. However, rare deletion burden has been shown to have a more deleterious effect in some studies in relation to IQ and components of cognition such as phonological working memory and selective attention [10, 11].

A recent meta-analysis of nine studies in clinical and population-based cohorts showed no association of CNV burden with intelligence quotient (IQ) [5]. Nor did they find association of burden and IQ within a subsequent analysis of a large cohort of psychosis patients and family members. Noteworthy, many of the clinical cohort studies included in the meta-analysis did show effects of rare CNV burden on cognition, although others have reported variable results [5, 12, 13]. Some of the discrepancies may be accounted for by differences between studies in the definition of rare CNV. Studies using a frequency cut-off of 1% for rare CNVs have reported associations more often than those using a 5% frequency cut-off [8–10]. Inadequate power and small sample size, particularly in clinical cohorts is also a factor.

An alternative to CNV burden is the use of constraint scores, e.g., “probability of being loss of-function-intolerant” (pLI). The pLI score classifies genes within CNV as being loss of function-intolerant, and the sum of all pLI scores of genes in a deletion are used to estimate the effect size of deletions on IQ [11]. In contrast to many CNV burden studies, these studies suggest that there is an impact of CNVs on cognition. Studies showed effects of deletions on IQ [11], and demonstrated a 3:1 effect size for of deletions and duplications on non-verbal IQ (NVIQ) [14]. Moreover, similar effect sizes were observed for deletions and duplications on IQ in unselected or general populations and autism spectrum disorders (ASD) cohorts [14]. Deleting 1 point of pLI had the same effect on NVIQ scores in both autism and population cohorts, indicating that CNV deletions do not differentially impact cognition in clinically ascertained and general population cohorts [14]. Overall, these studies suggest an impact of CNVs in aggregate on cognition. Some studies have adopted a more targeted approach focused on lists of CNV implicated in neurodevelopmental and neuropsychiatric conditions to further understand their specific contribution to cognitive ability [5, 15].

## Rare neurodevelopmental and neuropsychiatric associated CNVs (ND-CNVs)

Many rare CNVs have been broadly linked to neurodevelopmental and neuropsychiatric conditions (ND-CNV) including autism, schizophrenia (SCZ) and intellectual disability (ID). Some studies have investigated a list of aggregated ND-CNV, while others have used a gene-first approach, most commonly within a subset of 12 recurrent ND-CNV found to be robustly associated with neurodevelopmental and neuropsychiatric conditions [16, 17]. ND-CNVs show incomplete penetrance and variable phenotypes. Understanding the broader cognitive impact of ND-CNVs, or whether there are genotype-specific cognitive profiles may inform genetic counselling and guidance for carriers and their family members regarding the potential cognitive impact. It may also improve the implementation of cognitive and educational supports for those that may have more complex needs [18]. This review aimed to summarize the current research on ND-CNV cognitive phenotypes with a focus on (1) cognitive impact of aggregated lists of ND-CNV in both general and clinical cohorts to provide a better understanding of their impact in these populations, and (2) genotype-first approaches to understand if there are specific cognitive impacts associated with 12 recurrent ND-CNVs (Supplementary Table 1) [17]. This set of 12 ND-CNV were the focus here as they are well defined and most frequently reported in the literature. The rarity of ND-CNV leads to challenges in conducting sufficiently powered studies, therefore these ND-CNV are more likely to have genetic-first studies of cognition than more recently identified ND-CNV.

The term “carrier” is typically used to describe people with a genetic variation who do not express an associated phenotype, however, for the purpose of this review we use the term to refer to anyone with an ND-CNV, regardless of clinical phenotype (i.e., both clinical and non-clinical cohorts, or probands and familial carriers for some studies).

## Methods

Pub-Med and Embase databases were searched for studies that assessed the cognitive impact of ND-CNV. Included were studies that investigated pre-defined lists of ND-CNV in aggregate that also included one or more of the 12 recurrent ND-CNV, and studies that evaluated the individual effects of 12 recurrent ND-CNV on cognitive phenotypes [17]. For the individual effects of ND-CNV, 22q11.2 deletion was subsequently excluded from the review as the cognitive phenotype has previously been reviewed and characterized [19]. The 16p11.2BP4-BP5 CNV was included as it is associated with neurodevelopmental and neuropsychiatric conditions [20].

Final searches were conducted between the 16th of November 2021 and 6th of December 2021 and included any publication date up until the date searches were completed (see Supplementary Methods for search terms). Studies assessing cognition were included if they reported cohort performance on at least one cognitive test spanning cognitive domains of executive function, attention, learning and memory, language, perceptual-motor function, social cognition, or at minimum a measurement of overall intellectual functioning such as Full-Scale IQ (FSIQ), Verbal IQ (VIQ) or Performance IQ (PIQ). Studies were included if they assessed cognition in ND-CNVs in either general population or clinical cohorts across all age ranges with at least 5 subjects. Multiple studies from the same consortium or patient registry were included if the study provided an extension to previously reported analyses, or if additional participants were included. Exclusion criteria were studies not published in English, systematic or narrative reviews, meta-analyses, case reports, studies that assessed cognition in animal models, and studies that did not report any cognitive assessment score.

**Fig. 1:**
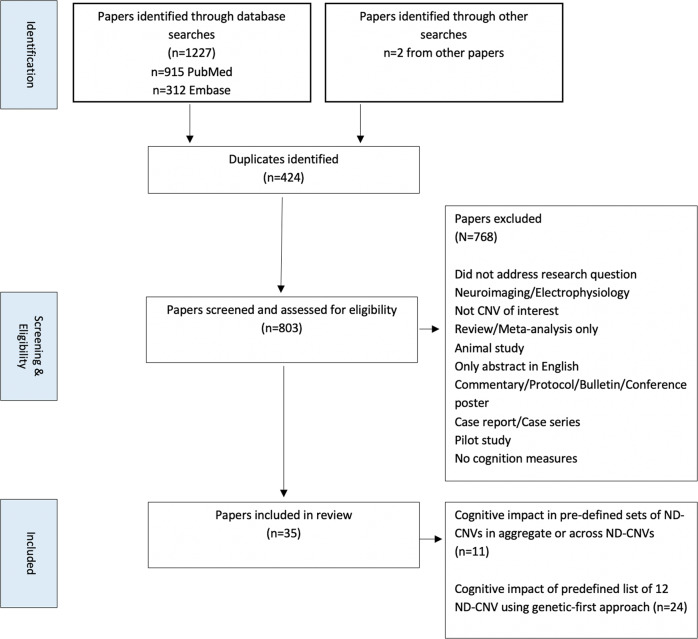
Flowchart of literature search and filtering of papers to be included in the final review. Papers were identified through database searches and then screened for eligibility for inclusion based on specific criteria.

We identified 1227 papers in total from database searches, and two further papers were identified from reference lists of the studies included, review papers, or from publication lists associated with Generation Scotland, UK Biobank (UKB), Simons VIP and IMAGEN datasets. After screening and filtering, 35 papers met criteria for inclusion in the review (Fig. 1).

### Cognitive impact of predefined sets of ND-CNVs in aggregate

Eleven studies investigated performance on a range of cognitive measures in pre-defined sets of ND-CNVs in aggregate (Table 1) [5, 10, 15, 18, 21–27]. Four studies were in general population cohorts [10, 15, 23, 24]; and seven were in clinically ascertained cohorts [5, 18, 21, 22, 25–27]. Each study investigated a slightly different ND-CNV list (see Supplementary Table 2 for full list for each study), with inclusion of at least one ND-CNV from the list of 12 ND-CNV subsequently reported in the genetic-first section below.

**Table 1:** Description of studies that assessed the aggregated effects of predefined lists of ND-CNV on cognition.

Paper	Cohort	Age mean (SD)*; range	CNVs analysed^a^	Genotype array	CNV calling	Cognition measures	Statistical analysis	Significant findings
Barone et al., (2021)	ASD cohort: ASD & CNV carrier (Causative-CNV *n* = 7; Non-causative CNV *n* = 18) ASD & non-carrier (*n* = 84)	Causative-CNV carrier: 7.57 yr. (2.93) Non-causative CNV carrier: 7.72 yr. (4.38) Non-carrier: 8.23 yr. (3.95)	9 causative- CNV 22 non-causative CNV	Human Genome array-CGH 8 x 60 K Microarray (Agilent)	ADM-2 algorithm	WISC-IV; Griffiths Mental Development Scale	Chi Square	• All causative-CNV carriers had IQ/GQ lower than 70. • No difference in rates of individuals with IQ/GQ less than 70 between all groups.
Bishop et al. (2017)	Clinical cohort: SSC ASD & dnLof or dnCNV carrier of high confidence ASD gene or locus (*n* = 112) ASD & non-carrier (*n* = 112)	ASD & dnLOF or dnCNV carrier: 113.10 mo. (39.75) ASD & non carrier: 112.83 mo. (75.46)	5 dnCNV 2 dnLOF	Illumina and WES	Unspecified	VIQ; NVIQ; PPVT-IV	Mixed model	• dnLoF or dnCNV carriers had greater mean VIQ compared to ASD non-carriers (pcorrected = 0.02); reduced NVIQ-VIQ difference (pcorrected = 0.01); and higher PPVT score (pcorrected = 0.01). • High within group variability for carriers of the same CNV.
Chawner et al., (2019)	Clinical cohort: IMAGINE-ID ND-CNV carriers (*n* = 258) Sibling comparison (*n* = 106)	ND-CNV carriers: 9.7 yr. (3.1) Sibling comparison: 10.9 yr. (3.0)	13 ND-CNVs	Unspecified, UK National Health Service	Unspecified	WASI; WCST (set-shifting); CANTAB: SWM, Spatial planning, Sustained attention, Processing speed	Linear mixed-effect models	• Poorer performance for ND-CNV carriers compared to non-carrier siblings for FSIQ (*p* < 1.00 × 10^−15^), PIQ (*p* < 1.00 × 10^−15^), VIQ (*p* < 1.00 × 10^−15^), SWM (*p* = 6.50 × 10^−8^), spatial planning (*p* = 4.08 × 10^−5^), set-shifting (*p* = 5.99 × 10^−10^), and processing speed (*p* = 0.00895). • Genotype accounted for 5-20% of variance in phenotypic traits. • Genotype predicted poorer FSIQ (*p* = 2.37 × 10^−6^), PIQ (*p* = 6.03 × 10^−7^), VIQ (*p* = 5 × 10^−4^), spatial planning (*p* = 0.0134), processing speed (*p* = 1.44 × 10^−3^), but not set-shifting, SWM or sustained attention.
Cunningham et al. (2021)	Clinical genetics cohort: ND-CNV carrier (*n* = 169) Sibling comparison (*n* = 72)	ND-CNV carrier: median = 8.88 yr. (7.12, 10.96); 6.02-14.81 yr. Sibling comparison: median = 10.41 yr. (8.80,12.36); 5.89-14.75 yr.	17 ND-CNV	Unspecified in-house microarray or clinical genetic report	Unspecified	WASI	ANOVA; hierarchical regression; mediation analysis.	• Compared to siblings, ND-CNV carriers had lower FSIQ, PIQ and VIQ (all *p* < 0.001). • Poorer coordination in children with ND-CNV was associated with lower FSIQ (*p* = 0.011), PIQ (*p* = 0.015) and VIQ (*p* = 0.036). • Coordination ability was a partial mediator of FSIQ, PIQ and VIQ scores.
Guyatt et al. (2018)	Gen pop cohort: ALSPAC (*n* = 6,807) SCZ-CNV carrier (*n* = 85)	8 yr.	12 SCZ-CNV 9/12 found in sample	Illumina HumanHap550- Quad platform	PennCNV, HumanHap550 array libraries	WISC-III; NWRT; TEACh: Opposite Worlds, Sky Search tests; DANVA: face recognition task	Correlation	• CNV associated with lower IQ (*p* = 2.36 × 10^−4^). • Dels associated with lower IQ (*p* = 1.52 × 10^−4^), phonological memory (*p* = 6.46 × 10^−4^) & social cognition (*p* = 6.40 × 10^−4^). • No association between dups and any cognitive measure.
Hubbard et al., (2021)	Clinical cohort: CardiffCOGS SCZ & SCZ-CNV carrier (*n* = 15) SCZ non-carrier (*n* = 860) Control (*n* = 103) Clinical cohort: Irish Sample SCZ & SCZ-CNV carrier (*n* = 8) SCZ non-carrier (*n* = 511) Controls (*n* = 327)	CardiffCOGS SCZ-CNV carrier: 43.9 yr. (12.2) SCZ-CNV non-carrier: 43.4 yr. (11.9) Controls: age matched Irish Sample SCZ-CNV carrier: 43.7 yr. (9.5) SCZ CNV non-carrier: 41.2 yr. (12.4) Controls: age matched	12 SCZ-CNV	CardiffCOGS: HumanOmniExpressExome-8v1 (Combo array) Irish Sample: Affymetrix SNP Array 6.0 or Illumina HumanCoreExome (+custom) SNP array	PennCNV	MATRICS; WAIS III	Linear regression; fixed effects meta-analysis	• SCZ-CNV carriers had lower general cognitive ability than non-schizophrenia CNV carriers in the CardiffCOGS sample (*p* = 0.047) and Irish sample (*p* = 0.025), and analysis of both samples together (*p* = 0.003). • CNVs hitting loss of function intolerant genes were associated with lower cognition (*p* = 0.048).
Jensen et al. (2020)	Clinical cohort: SSC (*n* = 2290) Pathogenic CNV (*n* = 81) dnLGD (*n* = 81) No variant (*n* = 1921)	Not reported	78 pathogenic CNV dnLGD	Unspecified microarray	Unspecified	FSIQ	Mann-Whitney Test	• Pathogenic CNVs carriers had lower FSIQ scores compared to individuals with no variant (*p* = 0.002). • Individulas with dnLGD had lower FSIQ compared to individuals with no variant (*p* = 0.031)/
Kendall et al. (2017)	Gen pop cohort: UK Biobank (*n* = 152,728) SCZ-CNV carrier (*n* = 1087) Other ND-CNV carrier (*n* = 484) SCZ (*n* = 507)	Range: 40-69 yr.	53 ND-CNV (12 SCZ-CNV and 41 other ND-CNV)	Axiom Array or BiLEVE Array	PennCNV	Pairs matching test; RT; Fluid intelligence test; Digit span test; DSST; TMT-A & B	Linear regression	• SCZ-CNV carriers had poorer performance compared to non-carriers on fluid intelligence (*p* = 5.29 × 10^−10^), pairs matching (*p* = 0.003), RT (*p* = 1.52 × 10^−13^), digit span (*p* = 1.8 × 10^−5^); symbol-digit substitution (*p* = 3.0 × 10^−6^) and TMT-B (*p* = 2.07 × 10^−8^) tasks. • ND-CNV carriers had poorer performance compared to non-carriers on fluid intelligence (*p* = 3.97 × 10^−10^), pairs matching (*p* = 3.2 × 10^−4^), and RT (*p* = 9.46 × 10^−16^) tasks. • CNV carriers had greater performance that SCZ cohort and poorer performance than non-carriers.
Kendall et al. (2019)	Gen pop cohort: UK Biobank (no NDC *n* = 420,247) ND-CNV-carriers (*n* = 15,954)	Range: 18–65 yr.	33 ND-CNV	Axiom Array or BiLEVE Array	PennCNV	Pairs matching test; RT; Fluid intelligence test; Digit span test; DSST; TMT-A & B	Linear regression	• 24/33 CNVs associated with poorer performance on one or more cognitive task or functioning measure. • Greatest cognitive impact in 16p11.2 dup & del, 16p11.2 distal del & 22q11.2 dup carriers (average effect size range = –0.32 to –0.41).
Stefansson et al. (2014)	Gen pop cohort: Icelandic (*N* = 101,655) ND-CNV carrier (*n* = 167) Other CNV carrier (*n* = 475) Non-carrier (*n* = 475) SCZ (*n* = 161)	Range: 18–65 yr.	26 ND-CNV	Illumina HumanHap (300, 370, 610, 1M, 2.5M) and Illumina Omni (670, 1M, 2.5M, Express) SNP arrays	PennCNV	WMS-III: Logical memory subtest; COWAT; Category naming test: animals; Stroop test; TMT-A & B; WCST; CANTAB: SWM, RVIP; WASI-I; ARHQ; AMHQ	Generalized least squares regression	• Cognitive performance of ND-CNV carriers was greater than SCZ group, and poorer than non-carrier group. Other CNV carriers had similar performance to non-carriers. • Poorer VIQ associated with 16p11.2 del (*p* = 5.90 × 10^−16^), 16p12.1 del (*p* = 8.30 × 10^−6^); 17p12 del (*p* = 2.30 × 10^−9^), and 17p12 dup (*p* = 8.10 × 10^−5^). • Poorer PIQ associated with 16p11.2 del (*p* = 1.30 × 10^−6^) and 16p13.11 dup (*p* = 9.30 × 10^−6^). • 15q11.2BP1-2 del associated with difficulties in reading (ARHQ; *p* = 0.00019) and mathematics (AMHQ; *p* = 2.30 × 10^−5^).
Thygesen et al. (2021)	Clinical cohort: PEIC (*n* = 5,597) SCZ-CNV carrier (*n* = 29) Non-carrier (*n* = 3399)	SCZ-CNV carrier: 39.9 (15.6) yr. Non-carrier: 43.7 (15.6)	27 SCZ-CNV	Affimatrix SNP Array 6.0	PennCNV and Affymetrix Power Tools	Block design, combined digit span, RAVLT immediate and delayed recall	Linear mixed model	• SCZ-CNV were associated with poorer RAVLT immediate (*p* = 0.0036) and delayed (*p* = 0.0115) recall scores, but not digit span.

*Where mean (SD) is not available, median (Q1, Q3) is reported for age.

*ALSPAC* Avon Longitudinal Study of Parents and Children, *AMHQ* Adult Mathematical History Questionnaire, *ARHQ* Adult Reading History Questionnaire, *ASD* autism spectrum disorder, *CANTAB* Cambridge Neuropsychological Test Automated Battery, *COWAT* Controlled Oral Word Association Test, *CNV* copy number variants, *DANVA* Diagnostic Analysis of Non-verbal Accuracy, *dnCNV* de novo Copy Number Variants, *dnLGD* de novo likely gene-disruptive variant, *dnLoF* de novo loss of function mutations, *DSST* Digit-Symbol Substitution Task, *IQ* Intelligence Quotient, *MATRICS* Measurement and Treatment Research to Improve Cognition in Schizophrenia Consensus Cognitive Battery, *ND* neurodevelopmental, *NDC* neurodevelopmental or neuropsychiatric condition, *NVIQ* Non-Verbal IQ, *NWRT* Non-word Repetition Task, *PPVT* Peabody Picture Vocabulary Test, *RAVLT* Rey Auditory Verbal Learning Test, *RT* Reaction Time, *RVIP* Rapid Visual Information Processing, *SCZ* schizophrenia, *SWM* Spatial Working Memory, *TEACh* Test of Everyday Attention for Children, *TMT* Trail Making Task, *VIQ* Verbal IQ, *WAIS* Wechsler Adult Intelligence Scale, *WASI* Wechsler Abbreviated Scale of Intelligence, *WCST* Wisconsin Card Sorting Test, *WISC* Wechsler Intelligence Scale, *WMS* Wechsler Memory Scale.

^a^For full list of ND-CNV for each study see Supplementary Table 2.

The cognitive impact of specific ND-CNV sets have been identified in population-based cohorts of varying size. Kendall et al. (2017) investigated 53 pathogenic CNVs, 12 SCZ-associated and 41 other ND-CNVs, in 152,728 individuals from the UKB and their effects on seven cognitive tests (reaction time, simple and complex processing speed, fluid intelligence, numeric working memory, and visual attention, and cognitive performance) [24]. Sample sizes and statistical power varied by cognitive sub-test. ND-CNV carriers had poorer performance compared with non-carriers overall and ND-CNV carriers had the poorest performance with largest effects observed on fluid intelligence. Performance in the ND-CNV carriers was intermediate between non-carriers and a subgroup of individuals with SCZ identified in the cohort. A further study in 500,000 UKB participants identified significant associations with at least one measure of cognition or general functioning in 24 of 33 ND-CNV analyzed individually [15]. CNV penetrance was associated with effects on cognition; 16p11.2 duplication and distal deletion carriers showed greatest impacts. Mirror effects on cognition for reciprocal deletions and duplications were not observed. Average effect sizes were modest but assume equal weighting of performance on all tasks, although the numbers completing each were variable. A study in the Icelandic population (*n* = 101,655) identified 176 carriers of one of 24 ND-CNV [23]. They showed significantly poorer performance on cognitive tasks (PIQ, VIQ, letter and category fluency, Trail Making performance, set-shifting ability, spatial working memory, rapid visual processing and logical memory task)^,^ although the effects on cognitive performance in a SCZ subgroup was greater. When the analyses were controlled for IQ, only global functioning was significantly different between the groups. No differences in cognitive performance were observed in carriers of other large CNV not associated with neurodevelopmental or neuropsychiatric conditions in comparison with controls. A final study of ND-CNVs (*n* = 29) found significant effects of deletions, but not duplications, on IQ, non-word repetition and social cognition [10]. These findings suggest a greater impact of deletions on cognition; however, multiple testing correction was not conducted, and sample sizes for individual tasks were small.

Cognitive impacts of ND-CNV have also been reported in clinically ascertained cohorts. Chawner et al. (2019) reported that carriers of one of 13 ND-CNVs (*n* = 238) had significant differences in cognitive measures and traits linked to neurodevelopmental conditions compared with non-carrier siblings (*n* = 106) [18]. The observed effects were robust to correction for multiple testing and controlling for IQ. Specifically, large effects were observed in FSIQ, PIQ, VIQ, and sustained attention. Effects on spatial working memory, spatial planning and set-shifting ability were moderate and were small for processing speed. Significant effects were mostly observed when deletion and duplication carriers were separately compared. High levels of traits linked to neurodevelopmental conditions were observed in all ND-CNV carriers, while the observed effects on cognitive traits were weaker and more variable. No dosage effects of ND-CNV were observed and there were no specific profiles associated with deletions and duplications. The qualitative and quantitative nature of CNV effects were explored by ranking mean z-scores for each trait supporting differences between genotypes. Genotype predicted 5%-20% of variance in phenotypic traits, and significantly predicted the observed effects on FSIQ, PIQ and VIQ scores, processing speed and spatial planning, but not sustained attention, spatial working memory and set-shifting.

Cunningham et al. (2021) found that poor motor co-ordination was highly prevalent in children with ND-CNVs and closely linked to lower intellectual function and increased likelihood of mental health conditions [26]. They reported an association between motor co-ordination and low FSIQ, PIQ and VIQ, and increased ADHD and ASD symptoms in children with ND-CNVs (*n* = 169) compared with their closest-in-age unaffected siblings (*n* = 72). Motor co-ordination ability was found to fully mediate anxiety symptoms and to partially mediate FSIQ, PIQ and VIQ scores and ADHD and ASD symptoms.

Two studies assessed cognitive impacts in autism cohorts. Jensen et al. (2020) investigated the effects of 78 pathogenic CNV and de novo likely-gene disruptive (LGD) variants in 2290 autistic individuals from the Simons Simplex Collection (SSC), with and without intellectual disability [22]. Carriers of CNV or LGD variants in both groups had lower IQ compared with non-carriers in the same groups. The likelihood of carrying a deleterious variant was reduced in individuals with an IQ over 100. They reported an average decrease of 12.8 IQ points in carriers of de novo variants in a set of 173 autism-associated genes compared with non-carriers indicating a role for these genes in both autism and IQ. Barone et al. (2021) compared IQ or general quotient (GQ) in three groups of autistic children; carriers of autism associated CNV (ASD-CNV) (*n* = 7) or a non-causative CNV (*n* = 18), and non-carriers (*n* = 84) [25]. There were no significant differences in rates ID or developmental delay between the three groups, although those with ASD-CNV had IQ or GQ less than 70. The results were likely impacted by the small samples size in ASD-CNV group.

Finally, two studies assessed SCZ-associated CNVs in SCZ cohorts. Hubbard et al. (2021) found that carriers of 12 SCZ-CNV had lower general cognitive ability than non-carriers in both a discovery (*n* = 15 CNV carrier and 860 non-carrier) and replication sample (*n* = 8 carrier and 511 non-carrier), although SCZ-CNV samples sizes were low in both datasets [21]. Rare CNVs impacting genes intolerant to loss of function were associated with greater effects on cognition than genes impacted by CNV duplications. Poorer performance in immediate and delayed recall tasks of memory has also been reported within carriers of one of a pre-defined list of 27 SCZ-CNV from a dataset of individuals with schizophrenia and relatives (*n* = 29), suggesting specific cognitive impacts of these SCZ-CNV [5].

Overall, the aggregate studies reviewed showed moderate impacts across a broad range of cognitive domains were observed in ND-CNV carriers compared to non-carriers in the general population [15], and to a lesser degree than in those with an NDC [10, 23]. Carriers of some ND-CNV with an NDC show poorer cognition than non-carriers with an NDC, but this is not the case for all ND-CNV [21, 28]. The identification of overlapping or distinct cognitive traits among ND-CNV may help to understand how the underlying mechanisms disrupted in ND-CNV carriers contribute to differences in neurodevelopment and phenotypic traits such as cognition.

### Cognitive outcomes in groups defined by ND-CNV

Two studies separately compared cognition in different ND-CNVs to non-ND-CNV carriers from the general population or the SSC. Bishop et al. (2017) analyzed five de novo ND-CNVs identified in five or more individuals including 16q11.2 deletions and duplications, 15q11.2–13 duplications, 1q21.1 duplications and 7q11.23 duplications and compared to the SSC [27]. NVIQ and VIQ scores were above the SSC mean in 1q21.1 duplication carriers, and lower in 15q11.2–13 duplication and 16p11.2 duplication and deletion carriers, suggesting these ND-CNVs impact cognition to a greater extent. However, extrapolation of specific phenotypic signatures was difficult as sample sizes were small and there was high variability within ND-CNV carrier groups.

Individual analysis of the cognitive phenotype of 11 ND-CNVs in comparison to non-carrier controls in a general population cohort showed that 16p11.2 deletions, 17p12 deletions and duplications, and 16p12.1 deletions were significantly associated with VIQ with large effect sizes, while 16p11.2 deletions and 16p13.11 duplications were significantly associated with PIQ [23]. Overall carriers of 16p11.2 deletions displayed greatest impact across cognitive tasks. 16p11.2 deletions and duplications were associated with spatial working memory performance, and 16p11.2 deletions were also associated with letter fluency, stroop task and preservative errors on the Wisconsin card sorting task (WCST). 16p11.2 deletions and 22q11.2 duplications were associated with category fluency performance. 15q11.2BP1–2 deletions were associated with difficulties in reading and mathematics, while effects on other cognitive measures were only modest in nature, particularly when IQ was controlled for. All cognitive measures were impacted in 22q11.2 duplication carriers, although not all were statistically significant. These two studies suggest cognitive differences in specific ND-CNV. In the next section we focus on genotype-first studies to elucidate any distinct cognitive impacts of specific ND-CNV.

### Cognitive impact of a predefined list of 12 ND-CNV using a genetic-first approach

We identified 24 papers using a genotype-first approach to characterize cognition one of 12 recurrent ND-CNVs (Table 2). No studies assessing 3q29 deletions or 16p13.11 duplications were found using our search criteria. Within the 12 pre-defined ND-CNVs, impacts on at least one measure of mean FSIQ, PIQ or VIQ score, or fluid intelligence were found in both unaffected and clinically ascertained cohorts [29–33]. Genetic-first studies of ND-CNV are discussed separately.

**Table 2:** Description of studies that assessed the effects of predefined list of ND-CNV on cognition using a genetic-first approach.

Reference	Cohorts	Genotype array	CNV calling	Age mean (SD); range	Cognition measures	Statistical analysis	Significant findings
1q21.1 Del/Dup (*N* = 3)							
Bernier et al. (2016)	Simons VIP & cascade testing 1q21.1 Del (*n* = 9 children, *n* = 10 adults); 1q21.1 Dup (*n* = 10 children, *n* = 9 adults); Familial non-carrier (*n* = 8 siblings *n* = 15 parents)	Custom oligonucleotide array, and 60 K or 180 K microarray (Agilent) or clinical genetic report	Unspecified	1q21.1 Del: *Children:* 7.2 yr. (5.1); *Adults*: 40.7 yr. (16.8) 1q21.1 Dup: *Children:* 6.1 yr. (4.1); *Adults:* 35.2 yr. (11.2) Non-carrier: S*iblings:* 5.8 yr. (4.0); *Parents:* 40.3 yr. (11.9)	MSEL; DAS-II; WASI; CTOPP-4	Univariate analysis; Tukey post-hoc test	• Lower VIQ (*p* = 0.001) and NVIQ (*p* = 0.003) in 1q21.1 Dup compared to non-carriers. • No difference in VIQ or NVIQ between 1q21.1 Del and non-carriers, or between 1q21.1 Del and 1q21.1 Dup carriers.
Linden et al. (2021)	Simons VIP (*n* = 51); Lausanne (*n* = 12); Cardiff (IMAGINE ID, ECHO, DEFINE projects; *n* = 60) 1q21.1 Distal Del (*n* = 51 children, *n* = 17 adults); 1q21.1 Distal Dup (*n* = 44 children, *n* = 11 adults); Familial controls (*n* = 70)	Unspecified: in-house genotyping or clinical genetic report	Unspecified	1q21.1 Del: *Children:* 8.3 yr. (3.9); *Adults:* 34.7 yr. (8.9) 1q21.1 Dup: *Children:* 8.4 yr. (3.7); *Adults:* 46.5 yr. (8.9) Familial controls: not reported	DAS; MSEL; WASI-II; CANTAB; WCST	Linear mixed-effect models; Tukey post-hoc test	Children: • Lower FSIQ (*p* = 0.002), PIQ (*p* < 0.001) and VIQ (p = 0.029), spatial planning (*p* = 0.011) and spatial working memory (*p* = 0.009) in 1q21.1 Del carriers compared to controls. • Lower FSIQ (*p* = 0.025), PIQ (*p* = 0.035), VIQ (*p* = 0.033), spatial working memory (*p* = 0.001) for Dup carriers compared to controls. • No difference in FSIQ, PIQ or VIQ between Del and Dup carriers. Reduced sustained attention in Del compared to Dup carriers (*p* = 0.02). Adults: • Lower FSIQ (*p* = 0.002), PIQ (*p* = 0.014) and VIQ (*p* = 0.025) in 1q21.1 Del carriers compared to controls. • No difference in FSIQ, PIQ or VIQ between Dup carriers and controls, or Dup and Del carriers.
Sonderby et al. (2021)	UkBiobank 1q21.1 Distal Del (max *n* = 119); 1q21.1 Distal Dup (max *n* = 186); Non-carriers (max *n* = 468,709)	Unspecfied	PennCNV50 and iPsychCNV	Not reported for full cognitive data sample	Pairs Matching; RT; Reasoning and Problem-Solving; Digit Span; DSST; TMT-A; TMT-B	T-test corrected for multiple comparison (p < 0.007)	• 1q21.1 Del carriers had poorer performance than controls on symbol digit substitution (*p* < 1.4 × 10^–4^), trail making B (p < 3.1 × 10^–5^) and pairs matching (*p* < 7.3 × 10^–5^). • 1q21.1 Dup carriers had poorer performance than controls on reaction time (*p* < 2.1 × 10^–3^) and the reasoning and problem-solving task (*p* < 5.3 × 10^–3^).
2p16.3 Del (NRXN1 Del) (*N* = 1)							
Alfieri et al. (2020)	NRXN1 Del (total *n* = 5, *n* = 4 exonic; *n* = 1 intronic)	Agilent Human CGH Microarray 60 K Kit and Illumina NextSeq550 system	ADM2 (Aberration Detection Method) algorithm	NRXN1 Del: 104.6 mo. (56.6); 49-183 mo.	WISC-IV; Leiter-3; GMDS-ER	N/A	• Below-average or lower limit of average cognitive level was found Del carriers.
7q11.23 Dup (*N* = 1)							
Mervis et al. (2015)	7q11.23 Dup (*n* = 63 children, *n* = 16 toddlers, *n* = 12 adults)	Genetic microarray; FISH or qPCR to confirm classic Dup	Unspecified	Toddlers: 28.93 mo. (8.35); 18.33–45.57 mo. Children: 8.80 yr. (3.68); 4.01-17.76 yr. Adults: 36.39 yr. (9.13); 27.47–61.05 yr.	DAS-II; MSEL; WASI; PPVT-4; WIAT-III; EVT-2	N/A	• Toddlers: Median overall cognitive ability in low-average range; expressive language poorer than receptive language. • Children: Median GCA, verbal, nonverbal and spatial reasoning, working memory and processing speed in low average range. • Adults: Median FSIQ and PIQ in the average range, and VIQ in low-average range.
15q11.2 BP1-BP2 Del (*N* = 2)							
Ulfarsson et al. (2017)	Icelandic gen pop (*n* = 160,000) 15q11.2 BP1–BP2 Del (*n* = 71); Non-carriers (*n* = 643)	Unspecified	Unspecified	Not reported for cognitive data groups Range = 18–65 yr.	ARHQ; AMHQ VIQ; PIQ; LM-I; LM-II; LF; CF; Stroop; TMT-A; TMT-B SWM; RVIP; GAF;	Fisher’s exact test; Spearman’s correlation	• Del confer high likelihood for dyslexia (odds ratio=3.0, *p* = 2.2 × 10^−4^) and dyscalculia (odds ratio = 3.4, *p* = 4.9 × 10^−5^). • Poorer reading (ARHQ; p = 1.5 × 10^−4^) & mathematical ability (AMHQ; *p* = 7.4 × 10^−7^) in Del carriers compared to non-carriers. • Similar cognitive profile for 15q11.2 Del carriers and non-carriers with dyslexia and dyscalculia (Spearman correlation=0.58; =0.042).
Woo et al. (2019)	15q11.2 Del carrier parents (*n* = 22); Non-carrier parents (*n* = 22) Lumosity platform Controls (*n* = 6530)	Unspecified clinical aCGH or	Taqman assay (Hs01476346_cn, Life Technologies) to confirm BP1-BP2	15q11.2 Del carrier parents: 39.7 yr. (9.6) Non-carrier parents: 42.6 yr. (9.0) Controls: Age matched	GR; AR; Digital symbol coding; Divided visual attention; Reverse memory span; Forward memory span; RPM; Go/No-Go; TMT-A; TMT-B	Paired t-test, corrected for multiple comparisons (Nyolt’s Method)	• Poorer performance in 15q11.2 Del carrier parents compared to population controls in AR (*p* = 1.3 × 10^−2^), reverse memory span (*p* = 1.2 × 10^−3^); slower to complete GR task (*p* = 6.3 × 10^−3^). • Similar PIQ for 15q11.2 Del carriers, non-carrier parents, and controls.
15q11.2 BP1-BP2 Dup/Del (*N* = 2)							
ENIGMA writing committee (2020)	UK Biobank 15q11.2 BP1–BP2 Del (max *n* = 1790); 15q11.2 BP1–BP2 Dup (max *n* = 2117); Non-carriers (max *n* = 468,709)	Unspecified	PennCNV, Birdseye version 1.5.5 (Birdsuite), iPsychCNV	Not reported for full cognitive data sample	Pairs Matching; RT; Fluid Intelligence; Digit Span; DSST; TMT-A; TMT-B	T-test, corrected for multiple comparison (p < 0.003); Linear regression	• Del carriers poorer than non-carriers on 5/7 tasks, including Reaction time (*p* = 2.5 × 10^−13^), Fluid intelligence (*p* = 5.3 × 10-^11^), Digit span (*p* = 0.001), Symbol substitution (*p* = 0.001), TMT-B (7.1 × 10^-6^). • No difference between Dup carriers and non-carriers. • Gene dosage effects for RT (*p* = 9.6 × 10^−6^), FI (*p* = 9.6 × 10^−6^), and TMT-B (*p* = 0.002). •
Silva et al. (2021)	UK Biobank Total sample: 15q11.2 BP1-BP2 Del (max *n* = 1597); 15q11.2 BP1-BP2 Dup (max *n* = 1936); non-carrier (max *n* = 389,278) Neuroimaging: 15q11.2 BP1-BP2 Del (*n* = 102); 15q11.2 BP1-BP2 Dup (*n* = 113); non-carrier (*n* = 28,951)	See Kendall et al. (2017)	See Kendall et al. (2017)	Not reported for total cognitive data sample Neuroimaging sample: 15q11.2 BP1-BP2 Del: 55.4 (7.3); 40-68 yr. 15q11.2 BP1-BP2 Dup: 54.8 yr. (7.2); 40-69 yr. Non-carrier: 54.9 yr. (7.4); 40-70 yr.	Pairs matching; RT; Fluid intelligence; Digit span; DSST; TMT-A; TMT-B	ANOVA; post hoc pairwise comparisons; FDR correction	• In the total sample, excluding those with a neurological or psychiatric condition, Del carriers performed poorer than controls in RT (*p* = 4.20 × 10^−15^), pairs matching (*p* = 3.68 × 10^−5^), fluid intelligence (*p* = 6.37 × 10^−12^), digit span (*p* = 0.01), DSST (*p* = 0.004) and TMT-B (*p* = 4.37 × 10^−6^). Dup carriers performed poorer on pairs matching only compared to controls (*p* = 0.04). When including those with a neurological or psychiatric condition the same tasks were significant. • In neuroimaging sample, Del carriers performed poorer than controls in RT (*p* = 0.01), fluid intelligence (*p* = 0.01), DSST (*p* = 0.02), TMT-B (*p* = 0.02). No difference in Dup carriers compared to controls.
15q11.2-q13 dup (Dup15q) (*N* = 3)							
DiStefano et al. (2016)	National Dup15q Alliance, UCLA Dup15q Clinic & Centre for Autism Research and Treatment Dup15q (*n* = 13); Non-syndromic ASD (*n* = 13)	Unspecified clinical genetic report	Unspecified	Dup 15q: 69.15 mo. (42.22); 22-144 mo. ASD: 64.77 mo. (34.58); 22-125 mo.	MSEL; Leiter-R; SB-5; PLS-5	Independent samples t-test	• No difference in mean VDQ or NVDQ between isodicentric (*n* = 10) and interstitial (*n* = 3) Dups. • Lower VDQ in Dup15q with epilepsy (*n* = 4) compared to Dup15q without epilepsy (*n* = 8) (*p* = 0.007). • Dup15q and ASD were matched on DQ.
DiStefano et al. (2020)	National Dupl5q Alliance, UCLA Dupl5q clinic Interstitial Dup15q (*n* = 16); Isodicentric Dup15q (*n* = 46)	Unspecified clinical genetic report	Unspecified	Interstitial Dup15q: 85.88 mo. (40.48) Isodicentric Dup15q: 94.50 mo. (48.84) All participants: 30 mo.-18 yr.	DAS-II; MSEL	Correlation; Repeated-measures ANOVA; independent samples t-test; ANCOVA	• VDQ and NVDQ highly correlated across participants (*p* < 0.001). No difference between VDQ and NVDQ. • FSDQ in mild to severe ID range for majority of participants. • Isodicentric Dup had lower VDQ (*p* = 0.008) and NVDQ (*p* < 0.001) compared to interstitial Dup. • Isodicentric Dup without epilepsy had lower NVDQ compared to interstitial Dup (*p* = 0.04). • Isodicentric Dup with epilepsy had lower VDQ (*p* = 0.001) and NVDQ (*p* = 0.032) compared to isodicentric Dup without epilepsy.
Urraca et. al. (2013)	Interstitial Dup15q (*n* = 9)	Clinical genetics report and SignatureChipOS array (Signature Genomics) or Genome-Wide Affymetrix SNP 6.0 array	Unspecified	88.4 mo. (47.2); 3-16 yr.	WPPSI-IV; WASI	N/A	• Mean FSIQ was 80.8 ± 9.8 SD.
15q13.3 Del (*N* = 1)							
Ziats et al. (2016)	Baylor Medical Genetics Laboratory; Signature Genomics Laboratories 15q13.3 Del (total *n* = 18; *n* = 18 with BP4–BP5 Del, *n* = 3 with BP3–BP5 Del)	Unspecified chromosomal microarray for clinical genetics	Unspecified	15q13.3 Del: 14.0 yr. (1.7); 12-18 yr.	DAS-II	One sample t-test	• Mean FSIQ, VRIQ and NVRIQ all lower than mean normative score (all *p* < 0.001).
16p11.2 Del (*N* = 5)							
Hanson et al. (2010)	Children’s Hospital Boston; Tufts Medical Centre 16p11.2 BP4-BP5 Del (*n* = 11)	Oligonucleotide arrays (Agilent 244k, G4411B) or Affymetrix v6.0 array	Unspecified	Range = 3.25 -17.58 yr.	MSEL; DAS-II; WASI; CTOPP; PPVT-IV	N/A	• No individual scored in average range across all cognitive measures. NVIQ & VIQ scores were highly variable. • NVIQ was greater than VIQ in seven Del carriers.
Hanson et al. (2015)	Simons VIP 16p11.2 BP4-BP5 Del (*n* = 85); Familial non-carrier (*n* = 153)	Unspecified Clincial genetic testing	Unspecified	16p11.2 Del: IQ: 11.1 yr. (8.9) CASL: 9.6 yr. (3.8) CTOPP: 10.1 yr. (3.5) WIAT: 13.9 yr. (9.5) Non-carriers: IQ: 30.5 yr. (14.2) CASL: 10.6 yr. (4.2) CTOPP: 12.7 yr. (7.6)	MSEL; DAS-II; WASI; WIAT; CTOPP; NWRT; CASL	Linear mixed models	• Lower FSIQ, VIQ & NVIQ in 16p11.2 Del compared to familial non-carriers (all *p* < 0.001). • Overall CASL language score was lower in Del carriers compared to controls (*p* < 0.001), as well as CTOPP non-word repetition score (*p* < 0.001). • 67% of Del carriers performed ≥2 SD below WIAT word reading test mean; 32% performed ≥2 SD below WIAT numerical operations test mean.
Mei et al. (2018)	VCGS (Australia); Simons VIP (US) 16p11.2 Del carriers (including DOC2A and TBX6) Australian (*n* = 7 adults; *n* = 40 children); US (*n* = 7 children, *n* = 1 adult)	Unspecified clinical genetics	Unspecified	Children Australian cohort: 8.27 yr. (1.85) US cohort: 9.84 yr. (4.41) Range=2.11-17.9 yr. Adults Australian and US cohort: 39.85 yr. (9.10) Range=20.4-48.2 yr.	WASI-II; K-BIT-2; CELF-Preschool 2/4/5; PLS-5; PPVT-4; TROG-2; CTOPP-2: NWRT; WRAT-4	N/A	• In children, mean NVIQ and literacy scores were >1 SD below the normative mean. Expressive & receptive language were also lower than standard mean. • 80% of children had auditory short-term memory & 94% had phonological memory difficulties. • In adults NVIQ and literacy scores were 2 SD below the normative mean, and language scores were >1 SD below the mean.
Osorio et al. (2021)	Lausanne ASD (*n* = 121); 16p11.2 BP4-BP5 Del: (*n* = 17); Typically developing (TD) (*n* = 45)	Unspecified clinical genetics	Unspecified	ASD: 5.49 yr. (2.9); 2-12 yr. 16p11.2 Del: 6.48 yr. (1.5); 2-12 yr. TD: 5.85 yr. (2.0); 2-12 yr.	WPPSI-IV; WISC-V; MSEL	MANCOVA; regression	• 16p11.2 Del carriers had lower FSIQ compared to TD (*p* = 8.9 × 10^−9^), as well as VIQ and NVIQ (both *p* < 0.001). • 16p11.2 Del carriers had higher VIQ compared to ASD (<0.01). • ASD had lower FSIQ compared to TD (*p* = 4.7 × 10^−23^), as well as VIQ and NVIQ (*p* < 0.001).
Zufferey et al. (2012)	16p11.2 European Consortium; Simons VIP; research literature 16p11.2 BP4-BP5 Del carriers (*n* = 258; *n* = 71 with cog data); Non-carrier intra-familial controls (*n* = 68)	Unspecified oligonucleotide arrays or clinical referral or customNimblegen arrays	Unspecified	16p11.2 European Consortium: Probands: 10.7 yr. Carrier siblings: 13.9 yr. Carrier parents: 37.4 yr. Simons VIP: Probands: 8.2 yr. Carrier siblings: 8.7 yr. Carrier parents: 342.7 yr. Research literature: ID/DD carriers: 9.8 yr.	MSEL; DAS-II; WPPSI-III; WISC-IV; WAIS-III; WASI	T-test	• Lower mean FSIQ in de novo carriers compared with familial non-carriers (*p* = 3.96 × 10^−27^). • Mean VIQ lower than NVIQ in Del carriers (*p* = 0.02). • 20% of Del carriers met DSM-IV-TR criteria for ID.
16p11.2Dup/Del (*N* = 6)							
Blackmon et al. (2021)	Simons VIP 16p11.2 Del (*n* = 30); 16p11.2 Dup (*n* = 25); Non-carrier controls (n = 55)	Unspecified clinical genetics	Unspecified	16p11.2 Del: 14.7 yr. (9.8); 7-48 yr. 16p11.2 Del non-carrier controls: 16.6 yr. (10); 7–63 yr. 16p11.2 Dup: 28.4 yr. (15.1); 7–63 yr. 16p11.2 Dup non-carrier controls: 27.2 yr. (14.4); 7–63 yr.	WASI; CELF-4; CTOPP	T-Test	• Lower FSIQ for Del carriers compared to case-matched non-carriers (*p* = 0.000005). • Lower FSIQ for Dup carriers compared to case-matched non-carriers (*p* = 0.04). • No difference in IQ, CELF, or CTOPP scores between Dup and Del carriers.
Chawner et al. (2021)	ECHO; IMAGINE-ID; Belgrade University Children’s Hospital neurodevelopmental CNV cohort; International 22q11.2DS Brain and Behaviour Consortium; Centre for Autism Research at Children’s Hospital of Philadelphia, 16p11.2 European consortium; Simons VIP; AGP 16p11.2 Del (*n* = 82); 16p11.2 Dup (*n* = 50); 22q11.2 Del (*n* = 370); 22q11.2 Dup (*n* = 45); ASD (*n* = 2027)	Unspecified clinical genetics	Unspecified	16p11.2 Del: 9.6 yr. (3.7) 16p11.2 Dup: 10.8 yr. (6.7) 22q11.2 Del: 13.4 yr. (3.4) 22q11.2 Dup: 10.1 yr. (4.3) ASD: 9.1 yr. (4.9)	FSIQ; VIQ; PIQ	ANCOVA; MANCOVA; Tukey’s method post hoc test; Levene’s test	• 16p11.2 Dup carriers had lower FSIQ (*p* = 6.71 × 10^−4^) and PIQ (*p* = 7.8 × 10^−6^) compared to Del carriers. • 16p11.2 Dup carriers had lower FSIQ (*p* = 2.91 × 10^−6^), VIQ (*p* = 1.9 × 10^−2^) and PIQ (*p* = 1.42 × 10^−4)^ compared to 22q11.2 Dup carriers. • 16p11.2 Del carriers had lower FSIQ (*p* = 3.10 × 10^−8^), VIQ (*p* = 3.8 × 10^−2^) and PIQ (*p* = 1.9 × 10^−9^) compared to 22q11.2 Del carriers. • No difference in IQ between 16p11.2 Del and 22q11.2 Dup, or between 16p11.2 Dup and 22q11.2 Del. • More variable IQ for Dup compared to Del in both 16p11.2 (*p* = 0.001) and 22q11.2 (*p* < 0.001). • 16p11.2 Dup subgroup with ASD had lower PIQ compared to non-carrier ASD (*p* = 0.006). No difference in IQ between 16p11.2 Del with ASD and non-carrier ASD group. •
D’Angelo et al. (2016)	16p11.2 European Consortium; ECHO & Simons VIP (US) European: 16p11.2 Dup (*n* = 97 proband carriers; *n* = 6 paediatric carrier relatives; *n* = 24 adult carrier relatives); Non-carrier relatives (*n* = 12) 16p11.2 Del (*n* = 170 proband carriers; *n* = 21 paediatric carrier relatives; *n* = 31 adult carrier relatives); Non-carrier relatives (*n* = 33) US: 16p11.2 Dup (*n* = 83 proband carriers; *n* = 17 paediatric carrier relatives; *n* = 43 adult carrier relatives); Non-carrier relatives (*n* = 90) 16p11.2 Del (*n* = 147 proband carriers; *n* = 11 paediatric carrier relatives; *n* = 10 adult carrier relatives); Non-carrier relative (*n* = 211)	Unspecified clinical genetics	Unspecified	European: 16p11.2 Dup: Proband carriers: 24.2 yr. (21.9) Paediatric carrier relatives: 5.7 yr. (3.4) Adult carrier relatives: 41.9 yr. (12.0) Non-carriers: 28.8 yr. (14.8) 16p11.2 Del: Proband carriers: 16.5 yr. (15.9) Paediatric carrier relatives: 10.6 yr. (3.5) Adult carrier relatives: 38.1 yr. (8.9) Non-carriers: 30.5 yr. (16.2) US: 16p11.2 Dup: Proband carriers: 9.1 yr. (8.8) Paediatric carrier relatives: 7.3 yr. (4.4) Adult carrier relatives: 40.3 yr. (10.2) Non-carriers: 28.9 yr. (17.8) 16p11.2 Del: Proband carriers: 7.6 yr. (4.9) Paediatric carrier relatives: 8.6 yr. (3.9) Adult carrier relatives: 39.0 yr. (5.0) Non-carriers: 28.9 yr. (14.9)	DAS; MSEL-AGS Edition; WASI	Linear mixed models; Levene’s test	• Dup were estimated to decrease FSIQ, NVIQ and VIQ (*p* < 0.001). Dup carriers had lower FSIQ compared to non-carrier relatives (*p* < 0.001). Largest effect observed in probands. • Del carriers had lower FSIQ compared to non-carrier relatives (*p* < 0.001). • Variability in FSIQ was higher in Dup compared to Del carriers (*p* < 0.001). • FSIQ, NVIQ and VIQ were greater in de novo compared to inherited Del, but no difference in Dup carriers. • Effect sizes remained similar for both Del and Dup when controlled for ASD and seizures.
Hippolyte et al. (2016)	16p11.2 European Consortium 16p11.2 Del (*n* = 37 probands; *n* = 25 carrier relative); 16p11.2 Dup (*n* = 21 probands; *n* = 23 carrier relative); Intra-familial controls (*n* = 71)	Unspecified clinical genetics	Unspecified	16p11.2 Del: 21.7 yr. (15.4); 4.8-59 yr. 16p11.2 Dup: 28.9 yr. (16.8); 3.3-65 yr. Intra-familial controls: 34 yr. (15.3); 3.3-62 yr.	WISC-IV; WASI; DAS-II; CTOPP: NWRT; DNAB; PPVT-R; TROG-2; PC robbery; Collective spelling tracking; forward, reverse digit & spatial span; CVLT; ROCF; TOL; Stroop; Go/No-Go	Linear mixed models	• Del and Dup carriers had lower FSIQ and NVIQ compared to familial controls (all *p* < 0.0001). • Compared to familial controls, Del carriers had poorer phonological skill scores (NWRT *p* = 5.2 × 10^−8^; oromotor sequences *p* = 2.7 × 10^−6^; sentence repetition *p* = 1.1 × 10^−5^), word definition (*p* = 2.1 × 10^-5^), reading fluency (*p* = 4.1 × 10^−5^), and executive functioning (stroop task *p* = 3 × 10^-6^). • Dup carriers had greater verbal long-term memory compared with familial controls when adjusting for NVIQ, (encoding *p* = 0.0002; delayed recall *p* = 0.0001). • Del carriers had poorer performance than Dup carriers in NWRT (*p* = 0.002), oromotor sequences (*P* = 0.001), verbal short- term memory (p < 0.002); verbal long-term memory (encoding p = 7.2 × 10^−6^; delayed recall *p* = 5.9 × 10^−5^) and executive functioning (stroop task *p* = 4 × 10^−5^).
Hudac et al. (2020)	Simons VIP 16p11.2 Del (*n* = 96); 16p11.2 Dup (*n* = 77)	Illumina HumanOmniExpress microarray	Unspecified	16p11.2 Del: 10.46 yr. (9.32); 0.8-48 yr. 16p11.2 Dup: 24.99 yr. (19.30); 1.7-80.1 yr.	WASI; DAS-II; MSEL	One-way ANOVA; linear mixed-effects model	• Del carriers had lower VIQ than Dup carriers (*p* = 0.023). No difference in NVIQ. • In Del carriers, a higher number of perinatal events was associated with lower VIQ (*p* < 0.001) and NVIQ (p = 0.001). A higher number of rare CNVs was associated with lower VIQ (*p* = 0.002) and NVIQ (*p* = 0.006). • In Dup carriers, a higher number of perinatal events was associated with NVIQ (*p* = 0.006). A higher number of rare CNVs was associated with lower NVIQ (*p* = 0.028).
Kim et al. (2020)	Simons VIP 16p11.2 Del (*n* = 110); 16p11.2 Dup (*n* = 58)	Unspecified clinical genetics	Unspecified	16p11.2 Del: 101.6 mo. (49.7); 24-250 mo. 16p11.2 Dup: 99.6 mo. (62.8); 24-281 mo.	DAS-II; MSEL.	T-test; Chi-square tests; regression	• Dup carriers had lower NVIQ compared to Del carriers (*p* < 0.05). • NVIQ predicted lower functional communication in everyday settings in both Del and Dup carriers (both *p* < 0.05). • In language, Del carriers had lower syntactic scores compared to Dup carriers (*p* < 0.05), and higher percentage with syntactic delays (*p* < 0.05). No difference between groups in pragmatic or semantic scores. NVIQ was associated with most language scores in both verbal Del and Dup carriers (*p* < 0.05).

Research studies were found for eight out of eleven ND-CNV.

*AGP* Autism genome Project, *AMHQ* Adult Mathematical History Questionnaire, *AR* Arithmetic reasoning, *ARHQ* Adult Reading History Questionnaire, *ASD* Autism Spectrum Disorder, *BP* Breakpoint, *CANTAB* Cambridge Neuropsychological Test Automated Battery, *CASL* Comprehensive Assessment of Spoken Language, *CNV* copy number variants, *CELF* Clinical Evaluation of Language Fundamentals, *CF* Category Fluency, *CTOPP* Comprehensive Test of Phonological Processing, *CVLT* California Verbal Learning Test, *DAS* Differential Abilities Scale, *DD* Developmental Delay, *Del* Deletion, *DNAB* Developmental Neuropsychological Assessment Battery, *DSM* Diagnostic and Statistical Manual of Mental Disorders, *DSST* Digit-Symbol Substitution Task, *Dup* Duplication, *DQ* Developmental Quotient, *ECHO* Cardiff University Experiences of Children With Copy Number Variants Study, *EVT* Expressive Vocabulary Test, *FSIQ* Full Scale Intelligence Quotient, *GAF* global assessment of function, *GCA* General Conceptual Ability, *Gen pop* General Population, *GMDS-ER* Griffiths Mental Developmental Scales Extended Revised, *GR* Grammatical reasoning, *ID* Intellectual Disability, *Leiter-R* Leiter International Performance Scales-Revised, *LF* Letter Fluency, *LM* Logical Memory, *MSEL* Mullen Scales of Early Learning, *NVDQ* Non-verbal DQ, *NVIQ* Non-Verbal IQ, *NWRT* Non-word Repetition Task, *N/A* Not Applicable, *PIQ* Performance IQ, *PLS-5* Preschool Language Scales-Fifth Edition, *PPVT* Peabody Picture Vocabulary Test, *ROCF* Rey-Osterrieth Complex Figure Test, *RT* Reaction Time, *RVIP* Rapid Visual Information Processing, *SB* Stanford-Binet Intelligence Scale-Fifth Edition, *SCZ* Schizophrenia, *Simons VIP* Simons Variation in Individuals Project, *SWM* Spatial Working Memory, *TMT* Trail Making Task, *ToL* Tower of London Task, *TROG* Test for Reception of Grammer, *UCLA* University of California, Los Angeles, *VCGS* Australia Victorian Clinical Genetics Service, *VDQ* Verbal DQ, *VIQ* Verbal IQ, *WASI* Wechsler Abbreviated Scale of Intelligence, *WAIS* Wechsler Adult Intelligence Scale, *WCST* Wisconsin Card Sorting Test, *WIAT* Wechsler Individual Achievement Test, *WISC* Wechsler Intelligence Scale, *WPPSI* Wechsler Preschool and Primary Scale of Intelligence, *WRAT* Wide Ranging Achievement Test.

### 1q21.1 duplication and deletion

Two studies assessed cognition in 1q21.1 duplication and deletion carriers in clinically ascertained participants and familial carriers from the Simons Variation in Individuals Project (VIP) and Intellectual Disability and Mental Health: Assessing the Genomic Impact on Neurodevelopment (IMAGINE-ID) cohorts, and one from the UKB general population [29, 33, 34]. Study outcomes were not consistent. One study of clinically ascertained children found lower FSIQ, PIQ, and VIQ compared to familial non-carriers in association with 1q21.1 duplications or deletions [34]. Separately a small sample of clinically ascertained adults identified reduced FSIQ, PIQ and VIQ in deletion carriers [34]. In contrast, Bernier et al. (2016) found significantly lower VIQ and NVIQ in duplication carriers compared to familial non-carriers [29]. They found no difference between duplication and deletion carriers. Phonological processing was in the extremely low range in both carrier types [29]. The discrepancies may be related to different sample sizes. Both studies included the Simons VIP cohort, giving rise to potential for overlapping participants. Analysis of the UKB identified poorer performance, processing speed, executive functioning, and declarative memory for 1q21.1 deletion carriers, and in reaction time and reasoning and problem-solving tasks for duplication carriers compared to controls [33]. This suggests different in cognitive impacts between 1q21.1 deletions and duplications, and that they both impact cognition in the absence of neurodevelopmental conditions.

### 2p16.3 (NRXN1) deletion

One study assessed a small sample of five people with NRXN1 deletions and ID diagnosis and reported four participants with below average cognitive ability and one in the low average range. It is worth noting that different cognitive measures such as NVIQ, FSIQ or DQ are reported for each participant [35].

### 7q11.23 duplication

Variability in cognitive, language and academic abilities was observed in children and adult carriers of 7q11.23 microduplications [31]. This was a case-only descriptive study of children with classic 7q11.23 microduplication, ascertained clinically and two smaller groups of toddlers and adults identified by cascade testing. Key findings in the toddler group were low average cognition associated with relatively poorer expressive compared with receptive language. The school age children had lower general conceptual ability (GCA), verbal, spatial, and nonverbal reasoning compared with the normative mean, and receptive language and expressive vocabulary scores in the average range. Adult carriers had average FSIQ and PIQ and low average VIQ that was significantly discrepant from PIQ in over half of carriers. They had relative reductions in expressive language compared with receptive language scores.

### 15q11.2 BP1-BP2 deletion

Three studies in 15q11.2 BP1-BP2 deletion carriers showed impacts on mathematical skills and aspects of language [23, 36, 37]. Lower reading and mathematical ability were identified in carriers in a population cohort compared with non-carriers [23, 36]. In a study using a web-based tool, poorer neurocognitive performances on tasks of arithmetic reasoning and a reverse memory span task of working memory were identified in 15q11.2 BP1-BP2 deletion carrier parents compared with age, gender and education matched population controls [37].

In the UKB, two studies of 15q11.2 BP1-BP2 deletion carriers showed significantly poorer performance on either four or five out of seven measures including fluid intelligence, Trail Making B, reaction time, digit-symbol substitution tasks, and digit span, when compared to controls [38, 39]. Duplication carriers did not differ significantly to non-carriers on any cognitive measure in one study [38], and had poorer performance in a pairs matching task only in the second study [39]. Copy number dosage at 15q11.2 BP1-BP2 was observed to have significant effects on reaction time, fluid intelligence and Trail Making B [38]. Overall, findings show that 15q11.2 BP1-BP2 deletions, but not duplications, impact multiple aspects of cognition.

### 15q11.2-q13.1 duplication (Dup15q Syndrome)

15q11.2-q13.1 duplications are frequently associated with isodicentric or interstitial duplication subtypes. Genetic subtype and epilepsy were associated with impairments in verbal and non-verbal developmental quotient (VDQ/NVDQ) in a group of children with different subtypes [40]. Isodicentric duplications were associated with lower VDQ and NVDQ compared to interstitial duplications although VDQ differences were likely related to co-occurring epilepsy in the isodicentric subtype. A prior study of a small subset of this cohort found no differences between isodicentric (*n* = 10) and interstitial (*n* = 3) genetic subtypes, likely due to the small sample size [41]. Separately, FSIQ was reported to be the low to average range in a small sample of interstitial duplication carriers (*n* = 9) [42].

### 15q13.3 deletion

One case-only study of 18 adolescent 15q13.3 microdeletion carriers reported mean FSIQ, VIQ and NVIQ to be significantly lower than the normative mean, with no difference between VIQ and NVIQ [32]. There was high variability in FSIQ scores, and 5/18 individuals also had a second CNV of uncertain clinical significance.

### 16p11.2 deletion and duplication

Reduced cognitive ability in carriers of 16p11.2 deletions has been reported by five studies [30, 43–46]. Three of these included participants ascertained by the Simons VIP consortium, resulting in a high probability of overlapping samples [30, 44, 46]. An initial case-only study (*n* = 11) showed high variability in VIQ and NVIQ [43].

Reduced FSIQ was reported in probands and parent and sibling carriers of 16p11.2 deletions compared to non-carrier familial controls [46]. There was no significant difference between probands and relative carriers or between de novo or inherited deletion carriers. Carriers had significantly lower mean VIQ compared to NVIQ, and 20% met the DSM-IV-TR criteria for ID. Hanson et al. (2015) observed similar effects on FSIQ, VIQ and NVIQ in addition to poorer spoken language scores, word reading and numerical operations academic tasks [30]. Mean NVIQ significantly below the normative mean in children and adults with 16p11.2 deletions, and language impairments, e.g., reduced word reading, and spelling scores were also reported in a case only study [44]. 60% of children had expressive and receptive language impairments.

One study compared 16p11.2 deletion carriers (*n* = 17) with ASD (*n* = 121) and neurotypical (NT) comparison groups (*n* = 45) [45]. The cognitive phenotype in 16p11.2 deletion carriers was between ASD and NT groups. Mean VIQ was 10-points higher in carriers compared to the ASD group. Both 16p11.2 deletion carrier and ASD groups had significantly lower FSIQ compared to NT group.

Six studies assessed cognitive phenotypes in both 16p11.2 deletions and duplication carriers. Results of comparisons between deletion and duplication carriers were mixed. Four studies used SVIP cohort with potential for overlap in samples, however, any studies with different analysis approaches are included [47–50].

D’Angelo et al. (2016) found reduced mean FSIQ, NVIQ and VIQ in duplication carriers compared with controls, and reduced mean FSIQ in deletion carriers compared to controls, even when accounting for ASD and seizures [48]. There was greater variance in FSIQ and larger effects on FSIQ in duplication carriers compared with non-carrier familial controls. There were IQ differences between carriers of de novo versus inherited deletions, but not duplication carriers. However, the IQ of transmitting parents accounted for 36% of the variance in inherited duplication carriers. Expanding on this, Hippolyte et al. (2016) found lower scores on measures of language (non-word repetition, oromotor sequences, sentence repetition, lexical and written language skills) and executive function (verbal inhibition) in deletion carriers [49]. Duplication carriers performed better than familial controls on tasks of verbal long-term memory, such as encoding and delayed recall tasks, and on verbal long-term memory. Deletion carriers had poorer performance than duplication carriers on tasks of non-word repetition, oromotor sequences, verbal short- and long-term memory, and inhibition supporting the observation of distinct cognitive differences between 16p11.2 CNV sub-types.

Reports from other studies comparing IQ between deletion and duplication subtypes have been mixed. Two reported lower cognitive scores in duplication carriers [28, 50], one reported lower scores in deletion carriers [51], and one reported no significant difference between groups [47]. These discrepancies may be related to sample size or study exclusion criteria. For example, one study focused on deletion and duplication carriers from the SVIP cohort that also completed neuroimaging which excluded individuals with reduced language and cognitive abilities.

Although FSIQ did not differ between deletion and duplication carriers in one study, both groups had lower FSIQ compared to case-matched non-carrier controls, similar to previous findings [47]. Further, a study that reported lower VIQ in deletion carriers compared to duplication carriers found a higher number of additional rare CNVs was associated with lower VIQ and NVIQ in deletion carriers, and NVIQ in duplication carriers [51], suggesting additional genetic influences on cognitive abilities.

In contrast, another study reported lower NVIQ in duplication carriers compared to deletion carriers and showed that poorer cognition significantly predicted lower functional communication in both groups when including verbal and minimally verbal individuals [50]. Even here, excluded participants were often minimally verbal, had significantly lower IQ and were more likely to be diagnosed with ASD compared to those who were included, therefore, conclusions regarding effects of ASD and NVIQ on language are limited.

Finally, Chawner et al. (2021) compared 16p11.2 deletion and duplication carriers, 22q11.2 deletion and duplication carriers, and an ASD group from the IMAGINE-ID cohort [28]. Similar to Kim et al. (2021), 16p11.2 duplication and 22q11.2 deletion carriers had lower FSIQ, VIQ and PIQ relative to 16p11.2 deletion and 22q11.2 duplication carriers. Cognitive performance in duplication carriers was more variable than deletion carriers. 16p11.2 deletion carriers with ASD had similar cognitive impairment to the ASD group, whereas duplication carriers with ASD had a poorer PIQ compared to the ASD group.

Overall, 16p11.2 deletions impact cognition, however it will be important to elucidate reasons for the discrepancies between studies comparing 16p11.2 deletions and duplications, and whether they relate to participant ascertainment or inclusion criteria for each study, or other factors such as additional CNVs.

### Domain-specific cognitive phenotypes in carriers of specific ND-CNVs

While only a limited number of ND-CNV have been investigated using adequately powered studies, general impacts across multiple cognitive domains were reported, some ND-CNV appeared to show greater effect sizes than others for different cognitive tasks which may suggest that some cognitive domains are more affected in certain ND-CNVs [15, 18].

Reduced grammatical and numerical reasoning were reported in 15q11.2BP1-BP2 deletion carriers in the general population and parent carriers [23, 37], with similar patterns observed in those with a diagnosis of dyslexia and dyscalculia [36]. These cognitive impacts are supported by neuroimaging studies, with reduced grey matter volume in left fusiform gyrus observed in 15q11.2BP1-BP2 deletion carriers [36], dyslexia [52] and dyscalculia [53]. Reduced differences in functional activity between phonological & orthographic familiar words and unfamiliar word forms within the fusiform gyrus in 15q11.2BP1-BP2 deletion carriers also suggests a lack of differentiation between word and non-word forms [36], similar to that observed in dyslexia [54].

16p11.2 CNVs are considered highly penetrant, and carriers exhibit the greatest degree of global cognitive impact in general population studies [15, 23]. 16p11.2 deletions have also been associated with language impacts [30, 44, 49], with large effects observed on VIQ in the general population [23], and lower non-verbal ability with poorer expressive, receptive, lexical and written language and verbal memory observed in clinically ascertained individuals [30, 49]. Brain structural differences in 16p11.2 deletion carriers compared with non-carriers have also been shown in language and phonological processing regions such as the transverse superior and middle temporal gyri [55].

One study showed relative strengths in verbal long-term memory in 16p11.2 duplication carriers, however this is the only study that reported increased cognitive performance associated with an ND-CNV [49]. Replication in a separate cohort is necessary, as reduced cognitive ability has been reported in other studies [18, 28].

The literature to date is limited, and the lack of in-depth speech and language assessment across ND-CNVs makes it difficult to draw inference about specific language impacts. While the suggested broad impacts of language abilities across ND-CNV is supported by reports of speech and language delay or language difficulties in some ND-CNV [56–59], a comprehensive language characterization across ND-CNVs is needed to fully understand their impact.

### Cognitive impact of deletions and duplications at the same loci

Some ND-CNVs are associated with duplications and deletions at the same locus, with gene dosage effects reported for traits such as height, body mass index (BMI), and macrocephaly/microcephaly in 1q21.1 CNV carriers [29, 60], and BMI and macrocephaly/ microcephaly in 16p11.2 CNV carriers [55, 60]. While carriers of both 16p11.2 deletions and duplications showed cognitive impacts [18], only one study suggested potential gene-dosage effects, with greater verbal long-term memory in 16p11.2 duplications compared to deletion carriers and NVIQ matched controls [49]. However, this requires replication in a larger sample. As for 1q21.1 CNV carriers, cognitive reports are inconsistent in clinical cohorts of children and adults [18, 29, 34], with greater impacts associated with 1q21.1 deletion compared to duplication in the general population [15, 33]. Similarly, deletions at 15q11.2 BP1-BP2 locus affected multiple aspects of cognition, while duplications did not [36, 38]. Overall, gene dosage effects on cognitive phenotypes were not observed, although findings mostly support the greater impact of deletions compared to duplications which is likely explained by haploinsufficiency or loss of function having greater impact than gain of function.

### Influence of additional factors on variability in cognitive phenotypes

Chawner at al. (2019) showed that genotype accounted for between 5 and 20% of phenotypic variance, depending on the trait. Wide heterogeneity in cognitive ability within ND-CNVs reported in numerous studies implies the influence of other factors. For example, age effects have been reported, with increasing age associated with greater impairments in FSIQ and spatial working memory and reduced attention and set-shifting impairments in ND-CNV carriers [18]. Longitudinal studies are needed to accurately assess age effects on neurocognitive phenotypes in ND-CNV carriers throughout development. While sex effects have also been observed, with poorer performance on tasks of sustained attention and superior perceptual organization skills in males compared to females, sex accounted for a small amount of cognitive variation [18]. Many studies, especially genotype-first studies, may not be adequately powered to assess sex differences, leading to insufficient evidence of robust findings.

Physical traits such as motor difficulties are reported in many ND-CNV [18, 30], and may impact cognitive task performance [29]. While poorer coordination has been linked to reduced IQ in ND-CNV carriers [26], it could lead to reduced performance on certain tasks unrelated to an individual’s cognitive ability. Not all studies reviewed here assessed motor skills. However, motor skills should be considered when assessing performance on certain cognitive tasks.

When considering genetics, the size and location of a deletion or duplication within a CNV may contribute to the variability in cognitive phenotype observed between individual carriers of the same ND-CNV. For example, within the 15q11.2-q13.1 duplication (Dup15q), children with isodicentric duplications had lower VDQ and NVDQ compared to interstitial duplications, suggesting differential impacts [40]. Noteworthy, the isodicentric duplication often results in a triplication rather than a duplication leading to four copies and typically includes the 15q11.2 region, whereas the interstitial typically results in three copies of the 15q11.2-q13.1 region. While the information was not available for all studies, phenotypic heterogeneity is also potentially introduced through parent of origin and CNV coordinates as some may include the 15q11.2 BP1-BP2 region while others may include the 15q13.3 BP3-BP5 region. As for *NRXN1* deletions (2p16.3), exonic deletions are thought to be more clinically relevant than intronic deletions, with suggestions that exon 6–24 deletions are more penetrant with increased likelihood of developing ID and SCZ [61, 62]. While cognition has not been specifically compared across different deletions within the *NRXN1* gene, different cognitive impacts may also be present.

Apart from *NRXN1* deletions, the loci of the ND-CNV we reviewed affect multiple genes, many of which are highly expressed in the brain and associated with key functions, including synaptic formation and signalling [63]. A recent study linked genes within the 16p11.2 CNV locus and phenotypic traits, with the *SPN* gene shown to be associated with IQ and BMI, and the *YPEL3* gene with SCZ [64]. More in-depth analysis of genes and molecular mechanisms associated with ND-CNV phenotypes is required. While individual gene products may be affected by CNVs at specific locations, the potential downstream convergence on common mechanisms may also explain cross-genotype similarities in cognitive phenotypes.

Evidence for cognitive impacts of inheritance patterns remains unclear based on the reviewed articles [11, 46, 48], as many of the studies did not analyze the effect of inheritance status on cognitive outcomes. Further research is needed to examine suggestions that CNVs arising *de novo* are more deleterious, as it is thought they may negatively affect reproductive success and therefore have a reduced likelihood of being inherited [23]. For example, if two individuals have an identical CNV which is de novo in one person and inherited in the other, the CNV should have similar penetrance, although, the clinical and/or cognitive outcomes are likely influenced by other genetic or environmental factors. Another factor that hampers our understanding is that inheritance patterns are typically only available in clinical rather than population cohorts.

Quantifying the effect of ND-CNV in isolation is difficult, and cognitive phenotypes are additionally influenced by an individual’s genetic and environmental background. Family-based studies including parents and siblings may help to elucidate ND-CNV impacts relative to genetic background. Also, additional deleterious variants in the genetic background likely interact with ND-CNVs [65, 66]. A recent analysis 16p11.2 deletion carriers showed a correlation between FSIQ and number of additional rare variants [65], with a significant excess of additional variants also observed in 16p11.2 deletion proband carriers relative to carrier parents. To date, the cognitive impact of “other hits” has not been widely studied in the presence of ND-CNVs, although this is a growing area of research [66]. Further understanding of all possible genetic factors and their interactions is required to provide accurate prognostic information to ND-CNV carriers and families.

While we do not have sufficient evidence of impacts of environmental factors specifically in ND-CNV carriers, it is important to consider whether or how they cognitive or behavioural phenotypes, or later functional outcomes. Factors such as early life in utero exposures, birth traumas, or exposure to drugs during pregnancy may be important to consider although are often not systematically available in genetic cohorts. Future studies of ND-CNV carriers would benefit from the inclusion of psychosocial factors to evaluate their impact. In addition, more prevalent use of family-based study designs that are informative regarding inheritance may provide more insight into the heterogeneity of on cognitive and mental health outcomes.

### Functional outcomes and cognitive impact in ND-CNV

IQ is a predictor of many life outcomes, such as educational attainment, occupation, physical and mental health, and mortality [67–69]. Functional outcome impacts have been reported in ND-CNVs carriers in general or unselected population studies [15, 55]. Although fluid intelligence score altered the effect size of ND-CNVs on income, the low correlation between performance on certain cognitive tasks and functional outcomes suggests that cognition is not the only factor to consider. Other factors could influence functional outcomes, such as increased susceptibility for co-occurring medical conditions, which may impact educational opportunities or cognitive function [70]. In 15q11.2-q13.1 CNV carriers, cognitive ability was associated with poorer adaptive behaviour [40], suggesting impacts on daily living skills. Whether cognitive interventions help to improve adaptive behaviours and functional outcomes in ND-CNV carriers is yet to be evaluated.

### Limitations

Although the 12 ND-CNVs selected for review of genotype-first studies are based on a well-defined list [17], no genetic-first studies were identified for 3q29 deletions or 16p13.11 duplications. However, research in rare ND-CNV is evolving, such that, a study of cognition in 3q29 deletions was published following the literature searches conducted for this review. Klaiman et al. (2023) showed that participants with 3q29 deletions had a lower than average FSIQ overall, with over half showing a relative strength in verbal ability [71]. It is also worth noting that the of 12 ND-CNV is not exhaustive as suggested by the reviewed studies of aggregate ND-CNV (see supplementary Table 2 for full list of ND-CNV included in each study). As the ND-CNV field progresses review of genetic-first studies of other ND-CNV will be imperative to understand their cognitive impact.

The low prevalence of ND-CNVs often leads to small sample sizes and inadequate power to assess the impact of individual ND-CNVs on cognition. The probable sample overlap in studies including data from SVIP also affects interpretation of findings and cross-study comparisons. Sufficiently powered studies are necessary to understand ND-CNV-specific cognitive impacts and relative strengths, and replication of findings will be imperative to ensure robustness of findings.

Ascertainment bias, study criteria and statistical analysis should all be considered when interpreting discrepancies between studies. For example, general population samples, such as UKB, may have lower rates of neurodevelopmental or neuropsychiatric condition diagnoses or numbers of ND-CNV carriers due to enrolment criteria [15]. Conversely, clinically ascertained samples likely overestimate ND-CNV effects, as individuals are ascertained based on clinical phenotype and may have several other genetic and environmental factors contributing to cognitive phenotypes. Another consideration is the optimal comparison group to use. Non-carrier siblings have the advantage of partially controlling for shared background genetics, while general population controls do not [18]. Although closest in age sibling is often used, these studies are somewhat limited by the inability to strictly age and gender match. Choice of statistical model, variables and covariates may also affect comparison of findings between studies [72]. Some studies reviewed covaried for IQ in their analysis, however, this may overcorrect results as cognitive tasks are measuring processes which contribute to IQ [73].

Finally, different genotype arrays and CNV calling techniques have been used in the reviewed studies of aggregate ND-CNV. For the majority of genotype-first studies reviewed, the provision of a clinical genetic report is mentioned as inclusion criteria, but no details of genotype array or CNV calling are described. Differences in arrays and CNV calling algorithms used across the studies may contribute to the heterogeneity findings. Advances in whole genome sequencing and improved CNV calling methods are likely to lead in the future to more consistency across studies [74]. Additionally, WGS will provide more detailed information about the range of genetic variants contributing to outcomes in ND-CNV carriers.

### Future directions

Collaboration, registries, biobanks, and initiatives including the Genes to Mental Health Network (https://genes2mentalhealth.com/) are key for sufficient samples sizes to comprehensively characterize ND-CNV. Studies are currently underway for ND-CNV lacking in sufficient genotype-first cognitive phenotyping [75, 76]. Longitudinal studies will be important to understand the impacts of ND-CNV throughout the lifespan.

ND-CNVs are associated with heterogeneity in clinical features and cognitive ability, making it difficult to select appropriate measures to assess multiple cognitive domains across a wide range of abilities. The alignment research protocols and cognitive task batteries across studies would allow for collaboration and better comparison of similarities and differences between ND-CNV.

As ND-CNVs present with variable penetrance and phenotypic heterogeneity, a better understanding of the impacts of both common and rare genetic variance, gene-gene or gene-environment interactions, and environmental factors are required to elucidate their combined effects [65, 66].

## Conclusion

ND-CNV research is invaluable for understanding how underlying biological mechanisms are associated with neurocognitive and clinical outcomes. Greater knowledge of ND-CNV cognitive phenotypes may help the provision of appropriate clinical and educational supports to improve cognitive and functional outcomes for ND-CNV carriers who may have complex needs.

## Supplementary information

Supplemental
