# Supplementary material for: A review of the cognitive impact of neurodevelopmental and neuropsychiatric associated copy number variants
Source: Transl Psychiatry. 2023 Apr 8;13:116. doi: 10.1038/s41398-023-02421-6 (PMC10082763; doi:10.1038/s41398-023-02421-6)
Supplement: Supplementary file 1 — Supplemental [file 41398_2023_2421_MOESM1_ESM.docx]

Supplementary Methods

Database searches conducted to identify relevant studies and date search conducted:

- PubMed, (all fields): {Copy Number Variation OR CNV} AND {Cognition}; {Copy Number Variation OR CNV} AND {Intelligence}; {Copy Number Variation OR CNV} AND {IQ}; (16/11/2021).
- Embase, (title/abstract/author key words): {Copy Number Variation OR CNV} AND {Cognition}; {Copy Number Variation OR CNV} AND {Intelligence}; {Copy Number Variation OR CNV} AND {IQ}; (16/11/2021).
- PubMed, (all fields): {1q21.1 OR 1q21.1del OR 1q21.1 deletion} AND {Cognition}; {1q21.1 OR 1q21.1del OR 1q21.1 deletion} AND {Intelligence}; {1q21.1 OR 1q21.1del OR 1q21.1 deletion} AND {IQ}; (16/11/2021).
- PubMed, (all fields): {1q21.1 OR 1q21.1dup OR 1q21.1 duplication} AND {Cognition}; {1q21.1 OR 1q21.1dup OR 1q21.1 duplication} AND {Intelligence}; {1q21.1 OR 1q21.1dup OR 1q21.1 duplication} AND {IQ}; (16/11/2021)
- PubMed, (all fields): {3q29 OR 3q29del OR 3q29 deletion} AND {Cognition}; {3q29 OR 3q29del OR 3q29 deletion} AND {Intelligence}; {3q29 OR 3q29del OR 3q29 deletion} AND {IQ}; (17/11/2021).
- PubMed, (all fields): {15q11.2 OR 15q11.2del OR 15q11.2 deletion} AND {Cognition}; {15q11.2 OR 15q11.2del OR 15q11.2 deletion} AND {Intelligence}; {15q11.2 OR 15q11.2del OR 15q11.2 deletion} AND {IQ}; (06/12/2021).
- PubMed, (all fields): {15q13.3 OR 15q13.3del OR 15q13.3 deletion} AND {Cognition}; {15q13.3 OR 15q13.3del OR 15q13.3 deletion} AND {Intelligence}; {15q13.3 OR 15q13.3del OR 15q13.3 deletion} AND {IQ}; (06/12/2021).
- PubMed, (all fields): {16p13.11 OR 16p13.11dup OR 16p13.11 duplication} AND {Cognition}; {16p13.11 OR 16p13.11dup OR 16p13.11 duplication} AND {Intelligence}; {16p13.11 OR 16p13.11dup OR 16p13.11 duplication} AND {IQ}; (06/12/2021).
- PubMed, (all fields): {16p11.2 OR 16p11.2dup OR 16p11.2 duplication} AND {Cognition}; {16p11.2 OR 16p11.2dup OR 16p11.2 duplication} AND {Intelligence}; {16p11.2 OR 16p11.2dup OR 16p11.2 duplication} AND {IQ}; (06/12/2021).
- PubMed, (all fields): {16p11.2 OR 16p11.2del OR 16p11.2 deletion} AND {Cognition}; {16p11.2 OR 16p11.2del OR 16p11.2 deletion} AND {Intelligence}; {16p11.2 OR 16p11.2del OR 16p11.2 deletion} AND {IQ}; (06/12/2021).
- PubMed, (all fields): {Neurexin-1 OR NRXN1del OR Neurexin-1 deletion} AND {Cognition}; {Neurexin-1 OR NRXN1del OR Neurexin-1 deletion} AND {Intelligence}; {Neurexin-1 OR NRXN1del OR Neurexin-1 deletion} AND {IQ}; (17/11/2021).
- PubMed, (all fields): {7q11.23 OR 7q11.23dup OR 7q11.23 duplication OR WBS dup} AND {Cognition}; {7q11.23 OR 7q11.23dup OR 7q11.23 duplication OR WBS dup} AND {Cognition}; {7q11.23 OR 7q11.23dup OR 7q11.23 duplication OR WBS dup} AND {Cognition}; (17/11/2021).
- PubMed, (all fields): {15q11.2-q13.1 OR 15q11.2-q13.1dup OR 15q11.2-q13.1 duplication OR PWS/AS dup OR Dup15q syndrome} AND {Cognition}; {15q11.2-q13.1 OR 15q11.2-q13.1dup OR 15q11.2-q13.1 duplication OR PWS/AS dup OR Dup15q syndrome} AND {Intelligence}; {15q11.2-q13.1 OR 15q11.2-q13.1dup OR 15q11.2-q13.1 duplication OR PWS/AS dup OR Dup15q syndrome} AND {IQ}; (06/12/2021).

Supplementary Table 1. ND-CNV included in genotype-first literature searches. *ND-CNV returned no papers in searches. **ND-CNV subsequently excluded from review as it has been reviewed elsewhere.

| **CNV Included in Literature Search** |
| --- |
| 1q21.1 del  1q21.1 dup  Neurexin-1 exonic del (2p16.3)  3q29 del*  7q11.23 (WBS) dup  15q11.2 del  15q11.2-13.1 dup (PWS/AS dup or Dup15q)  15q13.3 del  16p13.11 dup*  16p11.2 dup  16p11.2 del  22q11.2 del** |

Supplementary Table 2. List of ND-CNV included in studies of aggregate analysis of ND-CNV effect on cognition. Each aggregate study also included one or more of the 12 recurrent ND-CNV investigated in the genotype-first section of the review.

| **Reference** | **List of ND-CNV included** |
| --- | --- |
| Guyatt et al. (2018) | 1q21.1 Del  1q21.1 Dup  2p16.3 Del  3q29 Del  7q11.23 Dup  15q11.2 Del  15q11.2-q13.1 Dup  15q13.3 Del  16p13.11 Dup  16p12.1 Del  16p11.2 Dup  22q11.2 Del |
| Bishop et al. (2018) | 15q11.2–13 Dup  16q11.2 Dup  DYRK1A LoF  CHD8 LoF  7q11.23 Dup  16p11.2 Del  1q21.1 Dup |
| Kendall et al. (2017) | SCZ-CNVs:  1q21.1 Del  1q21.1 Dup  NRXN1 Del  3q29 Del  WBS Dup  15q11.2 Del BP1-BP2  PWS/AS Dup  15q13.3 Del BP4-BP5  16p13.11Dup  16p12.1 Del (520kb)  16p11.2 Dup (593kb)  22q11.2 Del  Other ND-CNVs:  1p36 Del (GABRD)  1p36 Dup (GABRD)  TAR Del  TAR Dup  2q11.2 Del (LMAN2L, ARID5A)  2q13 Del  2q13 Dup  2q37 Del (HDAC4)  Wolf-Hirschhorn Del  Wolf-Hirschhorn Dup  Sotos syndrome Del  Williams-Beuren syndrome (WBS) Del  8p23.1 Del  8p23.1 Dup  9q34 Dup (EHMT1)  10q23 Del (NRG3, GRID1)  Potocki-Shaffer syndrome Del (EXT2)  15q11.2 Dup BP1-BP2  Prader-Willi syndrome/Angelman syndrome (PWS/AS) Del  15q24 Del  15q24 Del  15q25 Del  16p13.11 Del  16p11.2 distal Del (220kb)  16p11.2 distal Dup (220kb)  16p11.2 Del (593kb)  17p13.3 Del (YWHAE)  17P13.3 Del (YWHAE)  17.p13.3 Del (PAFAH1B1)  17p13.3 Dup (PAFAH1B1)  Smith-Magenis syndrome Del  Potocki-Lupski syndrome Dup  17q11.2 Del (NF1)  17q11.2 Dup (NF1)  Renal cysts and diabetes syndrome Del (RCAD)  17q12 Dup  17q21.31 Del  22q11.2 Dup  22q11.2 distal Del  22q11.2 distal Dup  SHANK3 Del  SHANK3 Dup |
| Steffansson et al. (2014) | 1q21.1 Del  1q21.1 Dup  2p25.3 (MYT1L) Dup  2p16.3 (NRXN1) Del  3q29 (DLG1) Del  7q36.3 (VIPR2) Del  7q36.3 (VIPR2) Dup  10q11.22-23 Del  10q11.22-23 Dup  13q31.3 (GPC6) Dup  15q11.2(BP1-BP2) Del  15q11.2-13.1(BP1- BP3) Dup  15q13.1(BP3-BP4) Del  15q13.1(BP3-BP4) Dup  15q13.3(BP4-BP5) (CHRNA7) Del  15q13.3(BP4.5-BP5) (CHRNA7) Del  16p13.11 (NDE1) Del  16p13.11 (NDE1) Dup  16p12.1 Del  16p11.2 dist (SH2B1) Del  16p11.2 Del  16p11.2 Dup  17p12 Del  17q12 Del  17q12 Dup  22q11.21 Del  22q11.21 Del  22q11.21 Dup  22q11.21 |
| Kendall et al. (2019) | TAR Del  TAR Dup  1q21.1 Del  1q21.1 Dup  NRXN1 Del  2q11.2 Del  2q13 Del (NPHP1)  2q13 Dup (NPHP1)  2q13 Del  2q13 Dup  2q21.1 Dup  10q11.21q11.23 Del  10q11.21q11.23 Dup  13q12 Del (CRYL1)  13q12.12 Del  13q12.12 Dup  15q11.2 Del  15q11.2 Dup  15q11q13 Dup breakpoints 3 and 4  15q13.3 Dup  15q13.3 Dup (CHRNA7)  16p13.11 Del  16p13.11 Dup  16p12.1 Del  16p12.1 Dup  16p11.2 Distal Del  16p11.2 Distal Dup  16p11.2 Del  16p11.2 Dup  17p12 Del (HNPP)  17p12 Dup (CMT1A)  17q12 Dup  22q11.2 Dup |
| Chawner et al. (2019) | 1q21.1 Dup  1q21.1 Distal Del  1q21.1 Distal Dup  2p16.3 Del  9q34.4 Del  15q11.2 Del  15q13.3 Del  15q13.3 Dup  16p11.2 Del  16p11.2 Dup  16p11.2 Distal Del  22q11.2 Del  22q11.2 Dup |
| Barone et al. (2021) | Causative CNV:  16p13.1 Del  16p12.2 Del  7q11.2 Del  12q21.2 Del  Xp22.3 Del  16p13.1 Dup  6q16.1 Dup  22q11.2 |
| Cunningham et al. (2021) | 15q11 BP2-BP3 Dup  15q11.2 Del  15q11.2 Dup  15q13.3 Del  15q13.3 Dup  16p11.2 Del  16p11.2 distal Del  16p11.2 Dup  1q21.1 Del  1q21.1 Dup  22q11.2 Del  22q11.2 distal Del  22q11.2 Dup  Kleefstra (9q.34.3 Del)  NRXN1 (2p16.3 Del)  TAR Del  TAR Dup |
| Hubbard et al. (2020) | 1q21.1 Del  1q21.1 Dup  NRXN1 exonic Del  3q29 Del  WBS Dup  15q11.2 Del  PWS/AS Dup  15q13.3 Del  16p13.11Dup  16p12.1 Del  16p11.2 Dup  22q11.2 Del  22q11.2 Dup |
| Thygesen et al. (2021) | \| 1q21.1.del \| \| --- \| \| 1q21.1.dup \| \| 2p25.3.dup \| \| 2p16.del \| \| 3q29.del \| \| 7q11.23 \| \| 7q36.3.del \| \| 7q36.3.dup \| \| 8q22.2 \| \| 9p24.3.del \| \| 9p24.3.dup \| \| 15q11.2.del \| \| 15q11.2-13.1.dup \| \| 15q13.3.I.del \| \| 15q13.3.II.del \| \| 16p13.11.dup \| \| 16p13.11.del \| \| 16p12.1.del \| \| 16p11.2.distal.del \| \| 16p11.2.del \| \| 16p11.2.dup \| \| 17p12.del \| \| 17q12.del \| \| 17q12.dup \| \| 22q11.21.large.del \| \| 22q11.21.del \| \| Xq28.distal.dup \| \|  \| |
| Jensen et al. (2020) | Not reported |
